# The Chromosome 9p21 CVD- and T2D-Associated Regions in a Norwegian Population (The HUNT2 Survey)

**DOI:** 10.1155/2015/164652

**Published:** 2015-05-18

**Authors:** Øyvind Helgeland, Jens K. Hertel, Anders Molven, Helge Ræder, Carl G. P. Platou, Kristian Midthjell, Kristian Hveem, Ottar Nygård, Pål R. Njølstad, Stefan Johansson

**Affiliations:** ^1^KG Jebsen Center for Diabetes Research, Department of Clinical Science, University of Bergen, 5021 Bergen, Norway; ^2^Department of Pediatrics, Haukeland University Hospital, 5021 Bergen, Norway; ^3^Morbid Obesity Center, Vestfold Hospital Trust, 3116 Tønsberg, Norway; ^4^Department of Heart Disease, Haukeland University Hospital, 5021 Bergen, Norway; ^5^Gade Laboratory for Pathology, Department of Clinical Medicine, University of Bergen, 5021 Bergen, Norway; ^6^Department of Pathology, Haukeland University Hospital, 5021 Bergen, Norway; ^7^HUNT Research Centre, Department of Public Health and General Practice, Norwegian University of Science and Technology, 7600 Levanger, Norway; ^8^Department of Internal Medicine, Levanger Hospital, Nord-Trøndelag Health Trust, 7600 Levanger, Norway; ^9^Center for Medical Genetics and Molecular Medicine, Haukeland University Hospital, 5021 Bergen, Norway

## Abstract

*Background.* Two adjacent regions upstream *CDKN2B* on chromosome 9p21 have been associated with type 2 diabetes (T2D) and progression of cardiovascular disease (CVD). The precise location and number of risk variants have not been completely delineated and a possible synergistic relationship between the adjacent regions is not fully addressed. By a population based cross-sectional case-control design, we genotyped 18 SNPs upstream of *CDKN2B* tagging 138 kb in and around two LD-blocks associated with CVD and T2D and investigated associations with T2D, angina pectoris (AP), myocardial infarction (MI), coronary heart disease (CHD; AP or AMI), and stroke using 5,564 subjects from HUNT2. *Results.* Single point and haplotype analysis showed evidence for only one common T2D risk haplotype (*rs10757282∣rs10811661*: OR = 1.19, *P* = 2.0 × 10^−3^) in the region. We confirmed the strong association between SNPs in the 60 kb CVD region with AP, MI, and CHD (*P* < 0.01). Conditioning on the lead SNPs in the region, we observed two suggestive independent single SNP association signals for MI, *rs2065501*  (*P* = 0.03) and *rs3217986*  (*P* = 0.04). *Conclusions.* We confirmed the association of known variants within the 9p21 interval with T2D and CHD. Our results further suggest that additional CHD susceptibility variants exist in this region.

## 1. Introduction

One interesting region associated with type 2 diabetes (T2D) and cardiovascular disease (CVD) is on chromosome 9p21 in a gene desert ~130 kb upstream of* CDKN2B*. Several SNPs in the 9p21 interval are strongly associated with MI [1–4], vascular disease [5–7], and cancer [8], all highly correlated (*r* ^2^ > 0.8) and to be found in a ~60 kb region in high linkage disequilibrium (LD). The 9p21 region also contains two adjacent, but separate, T2D signals; a strong signal mapped to a 2 kb LD-block (represented by* rs10811661* and* rs10757282*) and a putatively independent second signal (*rs564398*) located ~100 kb from the T2D interval [9–11].

After the initial genome-wide association studies (GWASs), several investigations confirmed the association with the 9p21 candidate SNPs in T2D [12–17] and CVD [18–24] and extended the number of CVD phenotypes associated with the region [25–30]. A shared mechanistic link might therefore exist within this region increasing risk of both CVD and T2D through a common pathway. In patients with T2D, a variant within 9p21 showed significant interaction between poor glycemic control and risk of angiographically verified coronary artery disease (CAD) [31]. However, the effects of the disease susceptibility variants for the two major disease loci have shown to be independent, since T2D risk variants do not seem to confer increased risk of cardiovascular disease or the other way around [5, 32].

A multilocus analysis of the 9p21 region suggested a haplotype-effect on T2D risk rather than an effect from one single SNP [33], indicating that the* bona fide* locus could be situated somewhere in the vicinity of the test SNPs. However, a comprehensive sequencing study of the 9p21 locus that assessed rare variants and their association with T2D and MI did not discover any variants with stronger association than what was found in the initial GWASs [8]. Thus, we chose to evaluate the distribution of common tagSNPs within the region in individuals with overlapping T2D, angina pectoris (AP), previous MI, or stroke from the Norwegian population-based HUNT2 survey to assess the distribution of T2D and CVD risk alleles in HUNT 2.

## 2. Materials and Methods

### 2.1. Study Subjects and Ethics Statement

**Figure 1:**
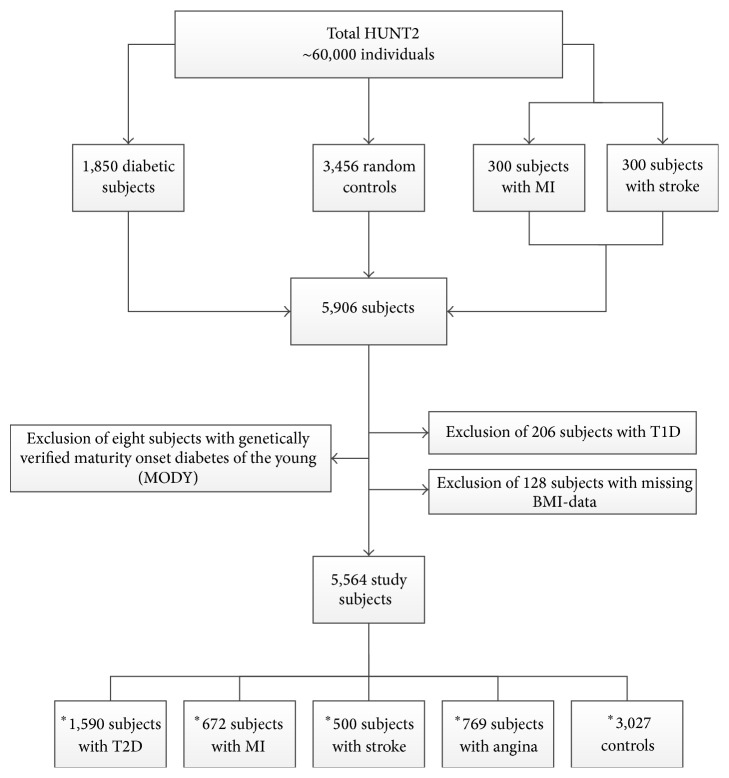
Flow chart presenting the selection of study subjects. Flow chart presenting the inclusion and exclusion criteria of the study subjects enrolled in the present study. A total of 5,564 subjects were eligible for analysis.  ^∗^Some individuals have more than one outcome (e.g., myocardial infarction (MI) and type 2 diabetes (T2D)); hence, the sum of these counts does not match the total counts of study subjects. T1D denotes type 1 diabetes. The final set of controls was reduced as subjects with MI and stroke were incorporated after the initial controls.

The second Nord-Trøndelag Health Study (HUNT2) is an extensive population-based health survey conducted in a Norwegian county with 127,000 inhabitants of which 60,000 participated [34]. HUNT2 is a subset of HUNT and was carried out in 1995–97. We had access to all subjects with diabetes (*n* = 1,850), in addition to 600 individuals selected for incident MI and/or stroke, but without diabetes, and 3,456 population-based random controls drawn from the same study population. After excluding 206 subjects with T1D, eight with genetically verified MODY [35], and 128 subjects with missing BMI data, 5,564 subjects were eligible for analysis (Figure 1). Diagnosis of diabetes, angina pectoris, previous MI, and stroke (ischemic or hemorrhagic strokes grouped as one phenotype) was self-reported. Written informed consent was obtained from all participants. This population based cross-sectional case-control study was approved by the Regional Committee for Research Ethics and the Norwegian Data Inspectorate, and was performed according to the latest version of the Helsinki Declaration.

### 2.2. SNP Selection, Genotyping, and Quality Control

We selected tagSNPs across 9p21 from the interval between Chr9:21,995,330 and 22,133,570 (NCBI Build 36). We selected 18 SNPs tagging a 138 kb region using the Haploview implementation of the Tagger algorithm [36] using the following criteria: minor allele frequency (MAF) of >5% and pairwise *r* ^2^ > 0.80. In addition, we added two previously GWAS-identified T2D susceptibility variants (*rs564398* and* rs10811661*) and three confirmed CVD susceptibility variants (*rs1333040*,* rs10757278,* and* rs1333049*). The genotyping was carried out by the multiplex MassARRAY* iPLEX* System (SEQUENOM Inc., San Diego, CA, USA) at CIGENE, Ås, Norway. Five variants (*rs1759417*,* rs1333049*,* rs7045889*,* rs4977761,* and* rs6475610*) did not pass quality control criteria (minimum call rate > 95% and Hardy-Weinberg equilibrium with *P* > 0.01) and were excluded from analyses. Thus, we assessed a total of 18 SNPs for association with T2D, angina pectoris, previous MI, and stroke.

### 2.3. Statistical Analysis

We used logistic regression to model single-point and haplotype association for the 18 SNPs with T2D, MI, angina pectoris, coronary heart disease, and stroke positive cases assuming additive effect of allele dosage. Gender, age, and BMI were used as covariates in the regression model in the analysis of T2D. Diabetes status and smoking were added to the list of covariates while analyzing AP, previous MI, CHD, and stroke. Individuals with a history of either AP, previous MI, or stroke were excluded as control subjects in the regression models when analyzing CVD traits. For T2D, AP, MI, and CHD, we carried out tests conditioning on the lead SNPs (MI, angina pectoris, CHD:* rs1333040* and* rs10757278*, T2D:* rs10811661*) to look for secondary signals of association. Multimarker haplotype analyses, haplotype frequency estimates, and haplotype comparisons for all phenotypes were performed using PLINK [37]. The sliding window approach used for multimarker haplotype analysis associates direct neighboring SNPs, generating 17 pairs of SNPs in the two-point analysis. All SNPs frequencies were consistent with Hardy-Weinberg equilibrium (HWE, *P* > 0.01). All analyses were carried out using PLINK version 1.07 software [37] and/or Stata SE v10.0 for Windows (Stata Corp LP, Brownsville, TX, USA). Figures displaying regional information such as the strength and extent of the association signals relative to genomic position, local linkage disequilibrium (LD), and recombination patterns and the positions of genes in the region were created using a combination of LocusZoom web interface [38], R package SNP Plotter [39], and Haploview [36]. We had >80% power to detect high-frequency alleles with ORs of 1.20 to 1.30 for both T2D and CVD phenotypes, but only around 50% and 30% power for T2D and CVD phenotypes, respectively, if the true ORs were 1.10. These estimates were performed using the Genetic Power Calculator [40]. All *P* values are presented without correction for multiple testing.

## 3. Results

Table 1 shows the clinical characteristics for the 5564 individuals enrolled in the present study.

### 3.1. Type 2 Diabetes

**Figure 2:**
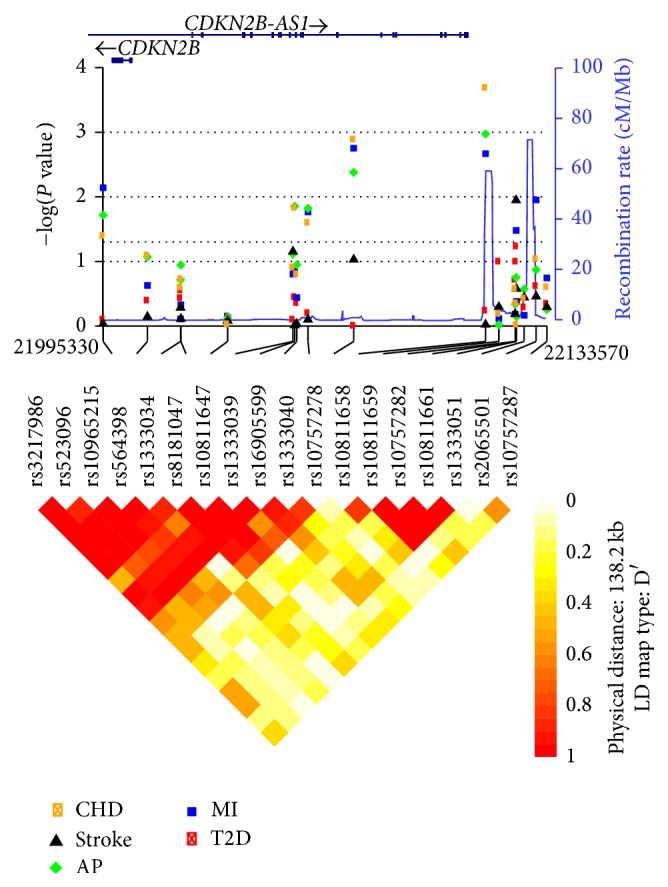
Plot summaries for single point association results. Plot summary of association results for 18 SNPs tagging the 138 kb CVD and T2D region on chromosome 9p21 for association with T2D, myocardial infarction, stroke, angina pectoris, or CHD (both MI and angina) using 5564 subjects from the HUNT2 study. The plot show local association results for all phenotypes together with the location and orientation of the genes it includes, local estimates of recombination rates and LD heat map with defined blocks (Gabriel et al.). The plots were created using the R-package SNP Plotter [39].

Regression analysis for association with T2D revealed only modest evidence for a single-point association for* rs10811661* (*P* = 0.058) after correction for age, gender, and BMI (Figure 2, Table 2). No SNP outside the previously implicated T2D block (LD-block 4 in Figure 2) showed evidence for an association with T2D.

Next, we performed a two-point sliding-window haplotype analysis and observed an increase in the association for this locus (*rs10757282*∣*rs10811661*) with T2D (*P* = 2.0 × 10^−3^) (Table 2). The association seemed to be driven by the C-T risk haplotype (OR = 1.19, *P* = 7.6 × 10^−4^), compared to the two other common two-marker haplotypes (Table 3). Further haplotype analysis in this LD-block revealed that* rs10757282* and* rs10811661* completely tagged one distinct risk haplotype spanning four consecutive markers in a 2-kb region (LD block 4 in Figure 2). We observed a breakup of the haplotype at markers* rs10811658* and* rs2065501*, which confines a candidate region, located 117–128 kb upstream of* CDKN2B*. The risk haplotype had a frequency of 29 versus 26% in cases and controls (Table 3). HapMap data indicated similar boundaries and frequencies for the haplotype (not shown). An exploratory analysis of increasing haplotype window sizes were performed but did only produce less significant results; the strongest association was found for haplotypes incorporating both* rs10757282* and* rs10811661*.

### 3.2. Cardiovascular Diseases: Angina Pectoris, Myocardial Infarction, and Stroke

Figure 2 and Additional file 1 (in Supplementary Material available online at http://dx.doi.org/10.1155/2014/164652) show the association results for each of the 18 SNPs with AP, previous MI, CHD (AP or previous MI), and stroke positive cases after adjustment for age, gender, BMI, diabetes status, and smoking. We report replication of the strong association between SNPs in the 60 kb CVD region (defined by* rs8181047* to* rs10757278*, Figure 2) with AP (*rs10757278*: OR = 1.22; *P* = 1.1 × 10^−3^, Figure 2), MI (rs1333040: OR = 1.23, *P* = 1.8 × 10^−3^, Figure 2), and CHD (*rs10757278*: OR = 1.37; *P* = 2.0 × 10^−4^, Figure 2). Subanalyses showed that the effect of the CHD-associated SNPs was strongest in those having the most severe phenotype including both AP and previous MI. None of the SNPs in the CVD region demonstrated association with stroke, but one marker (*rs10757282*) in the previously implicated T2D region did show nominal evidence for association with stroke (OR = 1.2 (1.04–1.38), *P* = 0.01, Figure 2).

In exploratory analysis, we observed several nominally significant potentially novel single SNP associations for angina pectoris, previous MI, individuals with both AP and previous MI, and stroke in the 138 kb interval (Additional file 1). After conditioning upon the highly confirmed CVD susceptibil0ity SNPs* rs1333040* and* rs10757278*, only two remaining SNPs (*rs206550*, OR = 1.32, *P* = 0.04; and* rs3217986*, OR = 1.15, *P* = 0.04) showed nominal *P* values <0.05 and only for MI (Table 4).

## 4. Discussion

Our findings highlight the genetic complexity of the chromosome 9p21 region. We found a weak but consistent single-point association between marker* rs10811661* and T2D, as previously found in several studies [9–11, 41]. This was in agreement with our former results obtained for this marker in a replication study performed in the same material from the HUNT2 population [13]. However, in the present study, we demonstrate a stronger association with a haplotype tagged by* rs10811661* and* rs10757282* and T2D. These results are in line with other studies [8]. Thus, these SNPs may tag a risk haplotype harboring an allele important for development of T2D. Alternatively, the 11 kb candidate region could harbor several variants associated with the disease.

Published data are conflicting regarding any additional T2D-associated signals in the 9p21 region [9–11]. Our data do not support the existence of additional signals. The role of* rs564398* as a T2D susceptibility variant is disputed [9, 12, 42]. Ethnicity may play a role, although our data are not supporting that this marker has a particularly strong effect in Caucasians [43].

The 9p21 risk variants are located in non-protein coding regions; their effects possibly influencing expression of nearby genes. The region contains two cyclin-dependent kinase inhibitors,* CDKN2A* (*p16* ^*INK4a*^) and* CDKN2B* (*p15* ^*INK4b*^), and CDKN2BAS, a large antisense noncoding RNA gene. Expression of these genes is coregulated and most of the confirmed CVD risk variants correlate with decreased expression of* CDKN2BAS* and furthermore to atherosclerosis [44, 45]. Recent follow-up studies show correlation between the number of risk alleles and atherosclerotic CAD progression, but no predisposition to MI in patients with preexisting atherosclerotic CAD nor increased reoccurrence of MI [46–48]. This suggests 9p21 risk variants promote atherosclerosis rather than triggering MI [49]. Our associations with angina pectoris as well as MI and with the strongest associations in those having both AP and previous MI at the time of screening may thus likely be mediated through increased propensity for atherosclerosis.

The* rs10757278* SNP has been highlighted as a potential functional variant for the association with atherosclerotic disease based on effects on expression of the* INK4*/*ARF* locus (p15^INK4b^, p16^INK4a^,* ARF* and* CDKN2BAS*) [50–52]. In the present study, we confirmed the associations for SNPs in the CVD region with AP and MI. The associations were strongest among subjects having both AP and previous MI. This could be a marker for early progression of atherosclerotic CAD, supporting the aforementioned association between 9p21 risk variants and early progression. Moreover, the* rs10757278* SNP has been mapped to one of 33 identified enhancers in the 9p21 interval, in which the risk variant disrupts a transcription factor binding site, which could have functional relevance for an atherosclerosis-associated pathway in human endothelial cells [53].

We found no association between SNPs in the CVD region and stroke. Our results are in accordance with some studies [5, 54], but not with others [52, 55]. Several investigations aiming to address this discrepancy have confirmed 9p21 as a risk factor for stroke, but with evidence for heterogeneity of effect across stroke subtypes. The strongest association has been shown for large vessel stroke [56]. Thus, lacking stroke subtyping in our study may be the reason we did not find this association. Participants of the HUNT2 survey were identified having stroke through a self-administered questionnaire, hence details regarding type of stroke, hemorrhagic versus ischemic, or subtypes like atherothrombotic or cardioembolic were not available. One could anticipate that SNPs in the 9p21 region associated with ischemic, but not hemorrhagic stroke. Studies have indicated that sequence variation in 9p21 influences atherosclerosis development and progression; the strongest association being seen for large vessels [29]. On the other hand,* rs1333040* has recently been linked to sporadic brain arteriovenous malformations known to increase hemorrhagic stroke risk [7]. Moreover, the adjacent* rs10757278* has been linked to hemorrhagic stroke [52]. These results might suggest different pathways for ischemic and hemorrhagic stroke sharing common mechanisms linked to the same SNPs in the 9p21 region. Interestingly, when restricting the analysis to subjects with T2D, several SNPs in the 60 kb CVD region appeared associated with stroke, with the most significant being* rs1333040* (OR = 1.44; *P* = 0.01). This association was not seen in stroke subjects without T2D. Interaction between variants within the 9p21 region and poor glycemic control increasing risk of CVD in patients with T2D has been suggested [31]. If similar associations were to be found for stroke risk in diabetics, it would be interesting to see whether poor glycemic control also affects different types of stroke differently.

Our exploratory results also highlights two potential novel CVD susceptibility variants,* rs3217986* and* rs2065501*, which are located close to, but not in strong LD with the former and well-confirmed CVD region. The* rs3217986* is located in the 3′ UTR of* CDKN2B* as well as in intron 1 of the non-protein coding* CDKN2B* antisense RNA,* CDKN2BAS*. Although speculative, it could be hypothesized that the risk variant of* rs3217986* might exert an effect on atherosclerotic CAD susceptibility by influencing expression of one or both of these two genes. To our knowledge, there are no reports on whether the risk variant of* rs3217986* is correlated with expression of* CDKN2B* and/or* CDKN2BAS;* thus, this hypothesis needs to be further resolved.

The study must be viewed in light of its limitations. Although previous studies have confirmed highly significant associations between SNPs in the region and CVD and T2D, the many tests performed in this study could lead to a risk of false positive findings. Thus, while the primary single SNP associations and the T2D-risk haplotype are supported by previous studies, the more explorative findings of putative secondary signals need to be further investigated in much larger cohorts. The sparse risk increase associated with these common variants also renders our findings inadequate for clinical prediction. Fine-mapping studies of disease associated regions may still prove important to guide further investigation towards understanding the disease pathogenesis and possibly providing tools for cost-efficient risk stratification in the future.

Despite the close proximity between the CVD and T2D risk regions, our study is in line with previous studies and indicates that there is no apparent overlap between the two risk regions. Theories with reference to the concrete disease mechanism mediated by the risk variants of the 9p21 interval have increased in numbers the last years. However, since most of them still remain exploratory, the exact nature of the disease associated variants and their targets require further elucidation. They may possibly differ between CVD and T2D. It is possible that large-scale genome sequencing efforts may aid by identifying the underlying risk variants in the 9p21 region.

## 5. Conclusions

In conclusion, we confirm the association between variants in the 9p21 interval with T2D and CHD. Our results suggest that there exist additional CVD susceptibility variants in this region, highlighting the genetic complexity of the 9p21 region and human disease.

## Supplementary Material

Sheet AP: Association results for angina pectoris (AP) from single and two-point haplotype analysis after correction for gender, age, BMI, diabetes and smoking.
Sheet MI: Association results for myocardial infarction (MI) from single and two-point haplotype analysis after correction for gender, age, BMI, diabetes and smoking.
Sheet MI+AP: Association results for both angina pectoris and myocardial infarction from single and two-point haplotype analysis after correction for gender, age, BMI, diabetes and smoking.
Sheet Stroke: Association results for stroke from single and two-point haplotype analysis after correction for gender, age, BMI, diabetes and smoking.

## Figures and Tables

**Table 1:** Clinical characteristic of the 5564 subjects included in the study and eligible for analysis.

	All	T2D	AP	MI	Stroke	No T2D and/or CVD
Individuals (*n*)	5,564	1,590^a^	769^a^	672^a^	500^a^	3,027^a^
Gender (male/female)	2,754/2,810	754/836	435/334	475/197	256/244	1,424/1,603
Age (years at examination)	60.4 ± 17.1	68.1 ± 12.0	72.4 ± 9.2	70.7 ± 10.3	70.8 ± 11.0	53.2 ± 17.6
BMI (kg/m^2^)	27.3 ± 4.4	29.2 ± 4.8	28.0 ± 4.3	27.5 ± 3.9	27.4 ± 3.9	26.4 ± 4.1
Ever smoked (yes/no)	2,600/2,964	647/943	345/424	367/305	241/259	1,468/1,559
Nonfasting serum glucose^b^ (mmol/L)	6.6 ± 3.1	9.6 ± 4.2	7.6 ± 3.6	7.2 ± 3.5	6.6 ± 2.7	5.4 ± 1.2
Serum triglyceride (mmol/L)	2.0 ± 1.3	2.5 ± 1.6	2.4 ± 1.6	2.3 ± 1.3	2.2 ± 1.5	1.8 ± 1.1
Serum cholesterol (mmol/L)	6.1 ± 1.3	6.2 ± 1.3	6.3 ± 1.3	6.2 ± 1.3	6.4 ± 1.3	6.0 ± 1.3
Serum HDL cholesterol (mmol/L)	1.3 ± 0.4	1.2 ± 0.4	1.2 ± 0.4	1.2 ± 0.4	1.3 ± 0.4	1.4 ± 0.4
Heart rate (bpm)	73.6 ± 13.6	75.5 ± 14.5	6.8 ± 13.6	67.6 ± 13.3	72.1 ± 13.4	74.1 ± 12.8
Type 2 diabetes (*n*, %)	1,590 (28.6%)	1,590 (100%)	326 (42.4%)	212 (31.5%)	110 (22%)	n/a
Myocardial infarction (*n*, %)	672 (12.1%)	212 (13.3%)	357 (46.9%)	672 (100%)	83 (16.6%)	n/a
Stroke (*n*, %)	500 (9.0%)	110 (6.9%)	115 (15.1%)	357 (53.1%)	500 (100%)	n/a
Angina pectoris (*n*, %)	769 (13.7%)	326 (20.5%)	769 (100%)	83 (12.4%)	115 (23%)	n/a

Values are presented as means ± SD or number (%). ^a^Some individuals have more than one outcome (for example MI + diabetes); hence, the sum of these column counts does not match the total counts of individuals. ^b^Only nonfasting glucose measures were available for participants in the HUNT2 cohort. MI denotes previous myocardial infarction. Abbreviations: T2D, Type 2 diabetes; AP, angina pectoris; MI, myocardial infarction; CVD, cardiovascular disease; bpm, beats per minute.

**Table 2:** Single point and two-point haplotype association results for T2D.

SNP	Minor allele	Single point		Two-point		Haplotype			
		OR (95% CI)	*P*	SNPs	Omnibus *P*	Haplotype	Frequency	OR	*P*
*rs3217986 *	C	1.03 (0.86–1.22)	7.74 × 10^−1^	*rs3217986*/*rs523096 *	4.69 × 10^−1^	AC/CT/AT	0.48/0.08/0.44	1.05/1.05/0.95	0.31/0.61/0.22
*rs523096 *	C	1.04 (0.95–1.14)	3.97 × 10^−1^	*rs523096*/*rs10965215 *	4.70 × 10^−1^	CA/TA/CG/TG	0.03/0.40/0.46/0.12	0.83/0.97/1.06/0.98	0.23/0.53/0.24/0.76
*rs10965215 *	A	0.96 (0.88–1.05)	3.63 × 10^−1^	*rs10965215*/*rs564398 *	5.64 × 10^−1^	GG/AA/GA	0.46/0.42/0.12	1.05/0.96/0.98	0.29/0.36/0.82
*rs564398 *	G	1.05 (0.96–1.15)	2.75 × 10^−1^	*rs564398*/*rs1333034 *	5.69 × 10^−1^	AG/GA/AA	0.12/0.46/0.43	0.97/1.05/0.96	0.71/0.29/0.41
*rs1333034 *	G	0.97 (0.84–1.12)	6.96 × 10^−1^	*rs1333034*/*rs8181047 *	9.32 × 10^−1^	AA/GG/AG	0.34/0.11/0.55	1.02/0.98/1.00	0.76/0.74/0.97
*rs8181047 *	A	1.01 (0.92–1.12)	7.78 × 10^−1^	*rs8181047*/*rs10811647 *	6.02 × 10^−1^	GG/AC/GC	0.40/0.34/0.26	0.96/1.01/1.04	0.35/0.78/0.44
*rs10811647 *	G	0.96 (0.87–1.05)	3.51 × 10^−1^	*rs10811647*/*rs1333039 *	6.49 × 10^−1^	CG/GC/CC	0.44/0.40/0.17	1.04/0.96/1.01	0.44/0.36/0.86
*rs1333039 *	G	1.04 (0.95–1.14)	4.35 × 10^−1^	*rs1333039*/*rs16905599 *	5.47 × 10^−1^	CA/GG/CG	0.06/0.44/0.50	1.05/1.04/0.95	0.62/0.40/0.28
*rs16905599 *	A	1.05 (0.87–1.26)	6.20 × 10^−1^	*rs16905599*/*rs1333040 *	9.28 × 10^−1^	AC/GC/GT	0.06/0.39/0.56	1.03/1.00/0.99	0.78/0.99/0.86
*rs1333040 *	C	1.00 (0.91–1.09)	9.72 × 10^−1^	*rs1333040*/*rs10757278 *	5.95 × 10^−1^	CG/TG/CA/TA	0.04/0.44/0.40/0.12	1.06/0.96/1.00/1.09	0.66/0.42/0.92/0.24
*rs10757278 *	G	0.97 (0.89–1.07)	5.67 × 10^−1^	*rs10757278*/*rs10811658 *	3.46 × 10^−1^	GA/AA/GG/AG	0.16/0.14/0.32/0.38	0.89/0.94/1.04/1.06	0.10/0.42/0.49/0.28
*rs10811658 *	A	0.92 (0.83–1.02)	9.85 × 10^−1^	*rs10811658*/*rs10811659 *	4.60 × 10^−1^	AC/GC/AT/GT	0.19/0.03/0.11/0.67	0.93/0.98/0.95/1.08	0.20/0.87/0.49/0.11
*rs10811659 *	C	0.93 (0.83–1.04)	1.89 × 10^−1^	*rs10811659*/*rs10757282 *	1.98 × 10^−1^	TC/CT/TT	0.44/0.21/0.35	1.07/0.92/0.98	0.14/0.14/0.72
*rs10757282 *	C	1.08 (0.99–1.18)	9.88 × 10^−2^	***rs10757282***/***rs10811661***	2.05 × 10^−3^	**CT/TT/CC**	**0.16/0.28/0.56**	**1.19/0.93/0.89**	7.63 × 10^−4^/0.11/5.71 × 10^−2^
*rs10811661 *	C	0.89 (0.78–1.00)	5.76 × 10^−2^	*rs10811661*/*rs1333051 *	8.58 × 10^−2^	CT/CA/TA	0.11/0.05/0.84	0.95/0.81/1.12	0.45/0.04/6.48 × 10^−2^
*rs1333051 *	T	0.95 (0.82–1.10)	5.04 × 10^−1^	*rs1333051*/*rs2065501 *	5.05 × 10^−1^	TA/AA/TC/AC	0.03/0.29/0.08/0.60	0.92/1.08/0.95/0.96	0.61/0.15/0.57/0.38
*rs2065501 *	A	1.06 (0.96–1.17)	2.36 × 10^−1^	*rs2065501*/*rs10757287 *	6.15 × 10^−1^	AT/CT/AA/CA	0.09/0.04/0.23/0.64	1.07/1.02/1.05/0.94	0.41/0.91/0.36/0.19
*rs10757287 *	T	1.05 (0.92–1.21)	4.43 × 10^−1^	n/a	n/a	n/a	n/a	n/a	n/a

Association results for T2D from single and two-point haplotype analysis after correction for gender, age, and BMI. Top associated haplotype *rs10757282* and *rs10811661* is outlined.

**Table 3:** T2D association results for haplotype *rs10757282*/*rs10811661*.

Haplotype	Frequency		OR	*P*
	Cases	Controls		
Overall evidence	—	—	—	2.05 × 10^−3^
CT	0.29	0.26	1.19	7.63 × 10^−4^
TT	0.56	0.57	0.93	1.06 × 10^−1^
CC	0.15	0.17	0.87	5.71 × 10^−2^

Association results for haplotypes defined by *rs10757282* and *rs10811661* in individuals with type 2 diabetes.

**Table 4:** Top five association results for CVD after conditioning upon lead SNPs.

SNP	Minor allele	AP		MI		Both MI and AP	
		OR (95% CI)	*P*	OR (95% CI)	*P*	OR (95% CI)	*P*
*rs3217986 *	C	1.21 (0.95–1.53)	0.13	1.32 (1.01–1.71)	0.04	1.25 (0.89–1.75)	0.19
*rs2065501 *	A	1.07 (0.94–1.21)	0.33	1.15 (1.01–1.32)	0.04	1.11 (0.94–1.33)	0.23
*rs10757282 *	C	1.09 (0.96–1.24)	0.18	1.14 (1.00–1.31)	0.05	n/a	n/a
*rs10811647 *	G	n/a		0.84 (0.69–1.03)	0.09	n/a	n/a
*rs16905599 *	A	1.25 (0.95–1.63)	0.11	1.27 (0.95–1.71)	0.10	1.31 (0.89–1.94)	0.17
*rs1333051 *	T	0.87 (0.71–1.07)	0.20	n/a	n/a	0.85 (0.64–1.13)	0.27
*rs8181047 *	A	n/a	n/a	n/a	n/a	1.16 (0.89–1.53)	0.28

Association results for the top five associated SNPs after conditioning upon the lead CVD SNPs *rs1333040* and *rs10757278* for individuals with angina pectoris (AP), myocardial infarction (MI), and both MI and AP.

## Abbreviations

AP: Angina pectoris
CAD: Coronary artery disease
CHD: Coronary heart disease
CVD: Cardiovascular disease
MI: Myocardial infarction
GWAS: Genome-wide association study
HUNT: Helseundersøkelsen Nord-Trøndelag
LD: Linkage disequilibrium
MODY: Maturity onset diabetes of the young
SNP: Single-nucleotide polymorphism
T2D: Type 2 diabetes
UTR: Untranslated region.
